# Safety and Tolerability of Mass Diethylcarbamazine and Albendazole Administration for the Elimination of Lymphatic Filariasis in Kenya: An Active Surveillance Study

**DOI:** 10.3390/ph14030264

**Published:** 2021-03-15

**Authors:** Christabel Khaemba, Abbie Barry, Wyckliff P. Omondi, Kefa Bota, Sultani Matendechero, Cecilia Wandera, Fred Siyoi, Elvis Kirui, Margaret Oluka, Pamela Nambwa, Parthasarathi Gurumurthy, Sammy M. Njenga, Anastacia Guantai, Eleni Aklillu

**Affiliations:** 1 Division of Clinical Pharmacology, Department of Laboratory Medicine, Karolinska Institutet, Karolinska University Hospital Huddinge, 14186 Stockholm, Sweden; cnkhaemba@gmail.com (C.K.); abbie.barry@ki.se (A.B.); 2 Pharmacy and Poisons Board, 27663-00506 Nairobi, Kenya; fmsiyoi@pharmacyboardkenya.org (F.S.); pmlnambwa@gmail.com (P.N.); 3 Ministry of Health -National Neglected Tropical Diseases Program, 30016-00100 Nairobi, Kenya; wyckliff.omondi@gmail.com (W.P.O.); kefabota@gmail.com (K.B.); hadleysultani@gmail.com (S.M.); cecienw@gmail.com (C.W.); 4 Ministry of Health -National Public Health Laboratory, Kenyatta National Hospital Grounds, 20750-00202 Nairobi, Kenya; elvokip@gmail.com; 5 Department of Pharmacology and Pharmacognosy, School of Pharmacy, University of Nairobi, 19676-00202 Nairobi, Kenya; olukamarga@yahoo.com (M.O.); anguantai@yahoo.com (A.G.); 6 Pharmacovigilance and Clinical Trials, Botswana Medicines Regulatory Authority, Gaborone 505155, Botswana; partha18@gmail.com; 7 Kenya Medical Research Institute (KEMRI), 54840-00200 Nairobi, Kenya; sammynjenga@gmail.com

**Keywords:** lymphatic filariasis, adverse events, mass drug administration, diethylcarbamazine, albendazole, Kenya

## Abstract

Preventive chemotherapy with diethylcarbamazine citrate (DEC) and albendazole (ALB) is the core intervention strategy to eliminate lymphatic filariasis (LF). We conducted a large-scale prospective active safety surveillance study to identify the incidence, type, severity, and risk factors for adverse events (AEs) following mass drug administration (MDA) of single-dose DEC and ALB in 10,010 participants from Kilifi County, Kenya. AEs were actively monitored and graded at 24 h, 48 h, and on day 7 Post-MDA. Out of 10,010 enrolled study participants, 1621 participants reported a total of 3102 AEs during a seven-day follow-up. The cumulative incidence of AEs was 16.2% (95% CI, 15.5–16.9%). The proportion of participants who experienced one, two, or ≥three types of AEs was 9.2%, 4.6%, 2.4%, respectively. AEs were mild (87.3%), moderate (12.4%), and severe (0.3%) and resolved within 72 h. The five most common AEs were dizziness (5.9%), headache (5.6%), loss of appetite (3.3%), fever (2.9%), and drowsiness (2.6%). Older age, taking concurrent medications, ≥three tablets of DEC, and type of meal taken before MDA were significant predictors of AEs. One in six participants experienced systemic mild-to-moderate severity grading and transient AEs. DEC and ALB co-administration for the elimination of LF is generally safe and well-tolerated.

## 1. Introduction

Lymphatic filariasis (LF), commonly known as elephantiasis, is a painful and disfiguring neglected tropical disease (NTD) caused by mosquito-borne thread-like filarial worms (nematodes); namely *Wuchereria bancrofti*, *Brugia malayi* and *Brugia timori* (*Brugia* spp.) [1] *Wuchereria bancrofti* accounts for over 90% of the LF infections. Adult filarial worms live in the lymphatic vessels and produce microfilariae (immature larvae) circulating in the peripheral blood. The infection is commonly acquired during childhood causing obscure damage to the lymphatic system. However, the painful and disfiguring visible manifestations of the disease, including lymphoedema, elephantiasis, and scrotal swelling, occur later in life, often leading to stigmatization and poverty [2,3,4]. According to the World Health Organization (WHO), LF is a leading cause of permanent and long-term disability, with more than 120 million people infected globally. The baseline data indicated that about 40% of LF infected people were from sub-Sharan Africa (SSA), with an estimated at-risk population of 432 million people [5]. Globally, 856 million people are at risk of LF, almost half of whom live in Africa, with 3.9 million of those in Kenya.

To alleviate LF-related disabilities and suffering, the fiftieth World Health Assembly passed a resolution in 1997 to eliminate lymphatic filariasis as a public health problem [6]. The Global Program to eliminate Lymphatic Filariasis (GPELF) was established in 2000 [7] with interruption of transmission through annual mass drug administration (MDA) of microfilaricidal drugs to the entire at-risk population being the core strategy [7,8]. Through the GPELF program 7.7 billion treatments were distributed to more than 910 million people at least once in 68 countries between 2002 and 2018 [4] Large-scale treatment (preventive chemotherapy) containing a combination of two antifilarial drugs, albendazole (ALB) with ivermectin (IVM) or diethylcarbamazine citrate (DEC) given annually is recommended by WHO to treat the entire at-risk populations for LF transmission [9]. The choice of drug combination for MDA depends on onchocerciasis co-endemicity. In areas where onchocerciasis is co-endemic, dual therapy consisting of ivermectin and albendazole (IA) is used, whereas in areas where onchocerciasis is not co-endemic, diethylcarbamazine citrate and albendazole (DA) are used [9]. Recently, triple therapy consisting of ivermectin, albendazole, and diethylcarbamazine citrate and (IDA) has been recommended for countries using DA to enhance the control and elimination of LF [9].

Large-scale MDA is delivered to all at-risk populations without prior screening or diagnosis, and the drugs are generally deemed safe. Most preventive chemotherapy uses drug combination, and the safety of the combined drugs needs to be ascertained. Given the relatively poor infrastructure in resource-limited regions, pharmacovigilance data involving MDA are scarce, particularly from SSA. Safety surveillance studies to detect the type, severity and risk factors of Adverse Events (AEs) are vital for careful planning and timely interventions. Previous studies reported that fear of AEs following MDA programs for elimination LF is a significant factor for poor MDA adherence [10,11]. Therefore, continued post-MDA surveillance is vital to boost public confidence in the program [12,13]. Immunologic reactions due to the parasites’ treatment-induced death among infected patients may also result in serious AEs (SAEs) [14,15,16]. The occurrence of SAEs highlights the benefits of having robust pharmacovigilance systems within the public health programs [9]. Indeed, the WHO recommends active surveillance for monitoring the safety of new drugs or interventions [9,17].

LF is found mainly in the coastal regions of Kenya along the Indian Ocean [18]. The Kenyan National Program to Eliminate Lymphatic Filariasis (NPELF) was established in 2001, and MDA with the standard dual therapy of DA was instituted in 2002. Due to financial and administrative challenges, the yearly MDA was not sustainable; however, with the support from the WHO, the annual MDA was resumed in 2015 [18]. However, the public health program mainly focuses on coverage, while less emphasis is given to safety monitoring.

The Kenya Pharmacy and Poisons Board (PPB) has a pharmacovigilance (PV) system to monitor safety signals through spontaneous reporting. However, there are minimal data on the safety of medicines used for MDA. Despite the comprehensive implementation of the LF elimination program, safety surveillance data on DA’s preventive chemotherapy are scarce, particularly from SSA, including Kenya. Locally collected drug safety information is vital for evidence-based decisions on public health interventions [17]. This study ‘s main objective was to identify the incidence, type, severity, and risk factors for adverse events associated with mass DEC and ALB administration for the control and elimination of LF in the coastal region of Kenya. Findings from this study will highlight the safety profile of drug combinations used in MDA to control LF in the community.

## 2. Results

### 2.1. Baseline Characteristics of the Study Population

**Figure 1:**
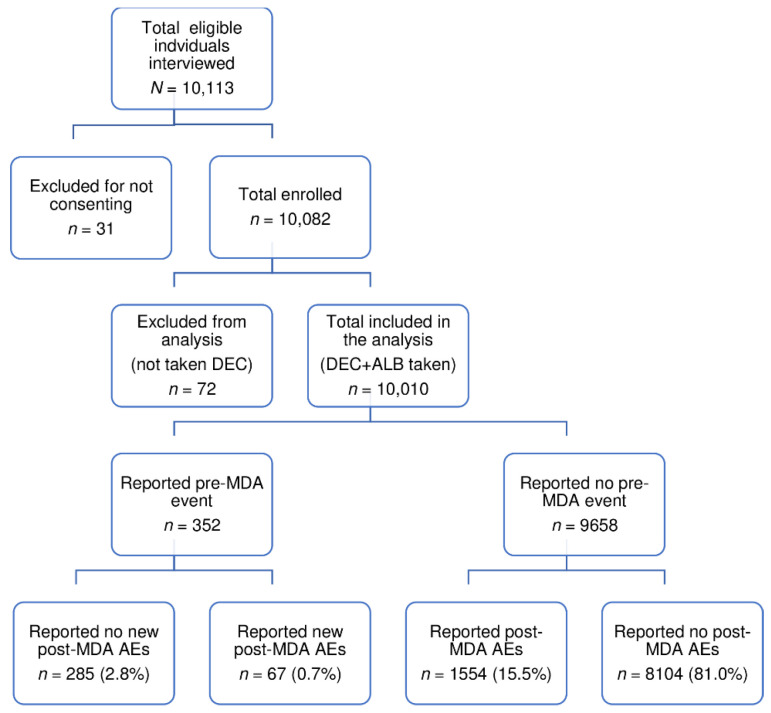
Study flow chart of participant enrolment and follow-up.

A convenience sample of 10,113 eligible individuals taking MDA was interviewed for enrolment during the 2018 National MDA campaign. However, 31 participants were excluded for not consenting, while 72 were excluded from the final analysis because they did not receive DEC. The study flow chart is presented in Figure 1.

Socio-demographic and baseline characteristics of study participants are presented in Table 1. Out of the total 10,010 study participants, 53.2 % were female, 44.52% were children under the age of 15, and 2.9% were 65 years and above. The study participants’ age ranged from 2–99 years, median age was 22 years, and Interquartile range (IQR) was 10–30 years. The median height was 140 cm (IQR 121–161; min 31, max 224), while the median weight was 46 kg (IQR = 26–58). Of the total study population, 63 % reported taking LF medication in the previous year; 0.82% reported having chronic medical conditions, with the most reported being hypertension, followed by asthma, chronic kidney disease, HIV, and diabetes. A total of 9296 (93.5%) of the participants reported sleeping under a mosquito net the previous night, 51.7% did not have screen windows, and 67.8% did not perform indoor spraying to prevent mosquito bites. (Table 1).

### 2.2. Incidence of Post-MDA Adverse Events

Out of the total 10,010 study participants, 1621 individuals reported one or more types of post-MDA AEs. The overall proportion of individuals who reported at least one type of post-MDA AEs over the 7-day follow-up period (cumulative incidence of any AEs) was 16.2% (95% CI; 15.5 to 0.16.9). A total of 3102 AEs was reported by 1621 participants over the 7-day follow-up period. The proportion of participants who reported one type of post-MDA event was 9.2% (*n* = 917). On the other hand, a total of 704 participants reported more than one type of AEs. The proportions of participants who reported one, two, three, and ≥4 types of AEs were 9.2% (*n* = 917), 4.6% (*n* = 464), 1.6% (*n* = 160) and 0.8% (*n* = 80), respectively.

### 2.3. Incidence and Types of Post-MDA Adverse Events

**Figure 2:**
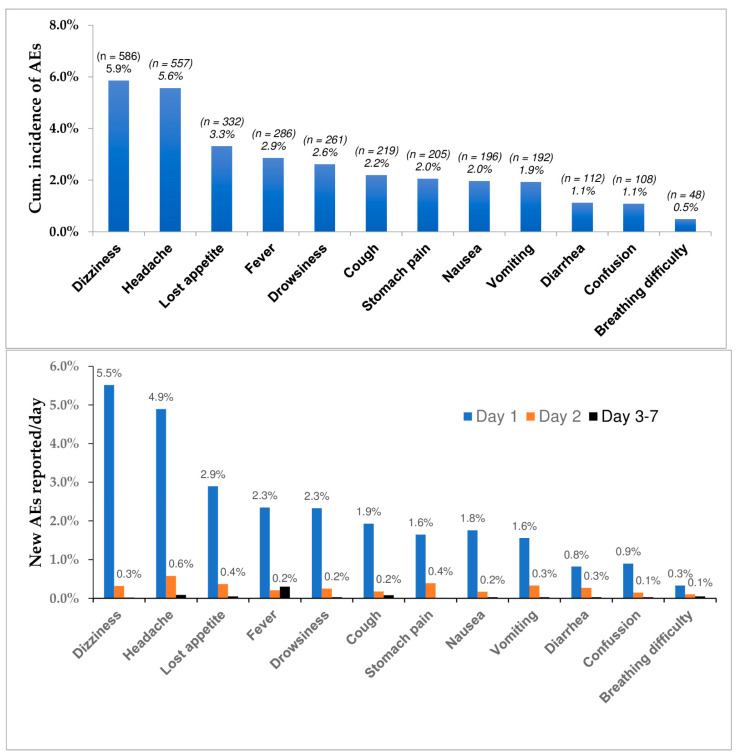
Cumulative incidence of post- mass drug administration (MDA) adverse events (AEs) over 7 days post-MDA (top), and incidence of new AEs reported per day (bottom) following mass diethylcarbamazine plus albendazole administration.

**Figure 3:**
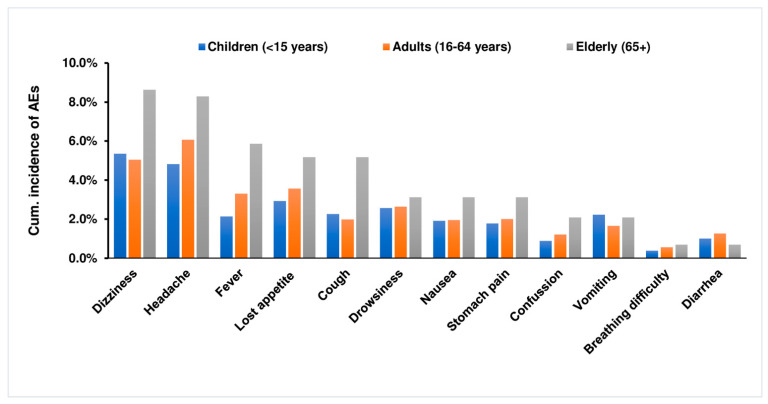
Cumulative incidence of adverse events (AEs) reported over 7 days following mass diethylcarbamazine plus albendazole administration among the different age groups.

The proportion of individuals who reported AEs during a seven-day follow-up period (cumulative incidence AEs) stratified by type of AEs, and incidence of new AEs reported per day is presented in Figure 2. The five most common post-MDA AEs were dizziness (5.9%), headache (5.6%), loss of appetite (3.3%), fever (2.9%), drowsiness 2.6%. Of the total adverse events reported, 26.9% (*n* = 2695) occurred on day one, 3.3% (*n* = 332) on day two and 0.7% (*n* = 73) on day 7. Dizziness (5.5%, *n* = 552) was the most reported AE on day one, followed by headache (4.9%, *n* = 490) and loss of appetite (2.9%, *n* = 290). All the reported events were transient and resolved within 72 h of occurrence (Figure 2). The cumulative incidence of adverse events reported during the 7-day follow up period among children (<15 years), adults (16–64 years) and elderlies is presented in Figure 3. While dizziness, headache, fever, and cough were more common in older people (65+ years) than in adults and children, vomiting was slightly higher in children and older people than in adults.

### 2.4. Severity Grading of Adverse Events

Out of the total 3102 AEs that were reported by 1621 participants over the seven-day follow-up period, 2865 events were graded for severity (Table 2). The observed AEs varied in severity, and no participant had a severity scale of more than Grade 3, and neither did any event require hospitalization. A total of 87.3% (*n* = 2502) of events were graded as mild (Grade 1), 12.4% (*n* = 354) were graded as moderate and 0.3% (*n* = 9) were graded as severe. Of the severe AEs, headache was the most reported.

### 2.5. Factors Correlated with Post-MDA Adverse Events

The incidence and correlations of post-MDA AEs are presented in Table 3**.** Though not statistically significant, female participants reported more AEs than male participants. Age group was significantly associated with the occurrence of AEs, being higher in the elderly ≥65 years (21.7%, *n* = 290) and 16–20 years (19.7%) age categories. Receiving a higher number of DEC tablets (≥3) was significantly correlated with the occurrence of AEs (*p* < 0.001). The incidence of AEs was highest amongst those who reported taking ≥three tablets of DEC (63.3%). Type of meal taken before MDA was also associated with AE occurrence. Participants who consumed a fatty or high protein meal before drug intake had a higher cumulative incidence of AEs (19.9%) compared to those who had a carbohydrate meal. The cumulative incidence of AEs among those who received MDA while on other concurrent medications was 25.9%. Of those who reported chronic illness, 24.4% of them experienced AEs.

### 2.6. Risk Factors Associated with Post-MDA Adverse Events

Univariate followed by a multivariate log-binomial regression analysis were conducted to identify risk factors for post-MDA AEs. In a univariate analysis, age category, concomitant medications, chronic illness, number of DEC tablets received, and type of meal taken before MDA were significant predictors of developing at least one type of post-MDA AE. In multivariate log-binomial regression analysis, age category, taking concomitant medication, tacking ≥3 DEC tablets, and type of meal taken before MDA remained significant predictors of post-MDA AEs (Table 4). Those who were elderly (≥65 years) and young adults (16–20 years) were at a higher risk of developing post-MDA AEs compared to a working-age group category (21 to 64 years). Having a fatty meal and high protein meal was a risk factor for developing post-MDA AEs compared to having a carbohydrate meal before drug intake.

## 3. Discussion

In this community-based active safety surveillance study, one in six individuals (16.2%) reported at least one type of adverse event following large-scale mass albendazole and diethylcarbamazine administration for the elimination of lymphatic filariasis. The incidence of experiencing one, two, and three or more types of AE simultaneously in the study population was 9.2%, 4.6%, and 2.4%, respectively. Most post-MDA AEs were mild (87.3%) or moderate (12.4%) and transient and resolved within 72 h of drug administration. The five most common AEs were dizziness (5.9%), headache (5.6%), loss of appetite (3.3%), fever (2.9%), and drowsiness (2.6%). Age category, co-medications, receiving ≥3 DEC tablets, and type of meal taken before MDA were significant predictors of AEs following MDA. To our knowledge, this is the first large sample size active cohort event monitoring study to investigate the prevalence, type, severity, and risk factors for AEs following mass ALB and DEC administration for the control and elimination of LF from sub-Saharan Africa.

Our study was well controlled to differentiate any pre-existing events from post-MDA AEs in each of the 10,010 study participants. Presence or absence of any event before drug intake, and if any, the type and severity were meticulously recorded and cross-checked for verification with any post-MDA reported event. Through an active door-to-door surveillance study, we quantified the AEs associated with DA and identified the types, frequency, and severity of AEs. Of the total study population, 16.2% reported at least one AE. Considering the number of AEs per respondent, 9.2% of the total study participants reported one event, 4.6% reported to have experienced two, and three AEs were reported by 1.6%. Previous studies from Sri Lanka, Brazil, India, Haiti, and a study by the WHO from 12 countries reported varying incidence of MDA-associated adverse events ranging from 12.6% to 61.1% [19,20,21]. Our finding is concurrent with previous studies showing that participants can experience more than one AE simultaneously [22].

A higher incidence of post-MDA AEs was observed in participants taking concomitant medication (25.9%) and who had a chronic illness (24.4%) It is well documented that drug interactions are one of the risk factors for the development of adverse events [23,24]. Accordingly, the incidence of post-MDA AEs in participants with chronic illness and taking concomitant medication could be due to drug–drug interaction or comorbidity. In this study, the type of concurrent medication or traditional medicine was not fully documented, which can be considered as our study limitation. This is an important aspect of safety monitoring that should be considered during future MDAs to ensure the minimization of drug-interaction-related AEs. Though not statistically significant, the incidence of post-MDA AEs was slightly higher in female compared to male participants (16.5% versus 15.9%). A previous study that investigated adverse reactions following mass drug administration with diethylcarbamazine in a total of 852 individuals from Brazil reported higher AEs in females than males [25]. This was also consistent with an observation conducted on a review of the literature following the mass drug administration for elimination of LF [26]. Sex-related physiological and hormonal differences that influence drug metabolism and disposition may be the reason for the observed higher incidence of AEs in women than men. Furthermore women are also more likely to pay attention to their health status and more often use medicines and therefore are more likely to report AEs compared to men [27].

The incidence of post-MDA AEs differed across the age groups. A higher incidence of AEs among participants aged 16–20 and above 65 years old was found compared to younger children and adults. It is well known that metabolic drug capacity is lower, and adverse drug reactions are common in older people [28,29]. Indeed, the association between a higher frequency of DEC-induced adverse reactions with increasing age has been reported previously [30]. However, the high incidence of AEs in the 16–20 years of age category may not be explained by variation in metabolic drug capacity. A recent study reported a high prevalence of LF infection in young adults and the elderly (65+ years) [31]. Previous studies reported significant side-effects often associated with the treatment of LF in infected patients due to treatment-induced immunologic reaction [14,15]. Though our study participants were not screened for LF, probably a high prevalence of LF infection in the two age categories mentioned above may explain the observed higher incidence of post-MDA AES due to immunologic reaction.

Dizziness, headache, loss of appetite, fever, and drowsiness were the most commonly reported adverse events in this study, which is consistent with reports from previous studies [10,20,21,26,32,33]. The reported AEs are similar to the summary of product characteristics for DEC and ALB [34,35]. Most of the reported AEs were mild ones that did not interfere with normal day to day activities. Of the reported severe AEs, headache had the highest frequency. These findings are consistent with previous studies that reported mild-to-moderate AEs following administration of DEC and ALB [32,33]. It is reported in the literature that systemic AEs (fever, headache, dizziness, nausea, and joint pains) occurring during the treatment of LF are due to the immunological reactions induced by the death of the LF parasite [14,15,16]. This could explain the type of AEs reported in our study with the assumption that those who experienced severe AEs to have a high load of microfilariae circulating in the blood; however, we cannot be conclusive as our study was performed in the general population without pre-screening for LF infection status. Interestingly, we found a positive association between the occurrence of post-MDA AEs with an increasing number of DEC tablets, which may indicate a dose-dependence effect.

Most the observed post-MDA AEs typically occurred within 24 h of drug administration and resolved within 72 h of occurrence. These findings are consistent with previous studies reporting the occurrence of AEs within 8 h of treatment, peaking at 12 h and 48 h and resolving within 7 days [25,26,30,32]. Given the transient nature of the AEs, the routine spontaneous reporting may result in underreporting of these events. This underscores the importance of integrating active surveillance methods of AE reporting in MDA. To our knowledge, no previous study explored any association between type of meal taken and the risk for AEs following MDA in the LF control program. Our result indicates that post-MDA AEs were more likely to occur in participants who had taken fatty or high protein meal than carbohydrates. This could be due to food–drug interactions, although we did not explore it further. Alteration of drug absorption and bioavailability by fatty, high protein, and fiber diets has been reported previously [36].

This study’s key strength is that it was an active surveillance study conducted using a large sample size. Therefore, results can be generalized to other LF endemic zones in the coastal region of Kenya and SSA at large. The burden of adverse reactions and management may affect the LF elimination program’s sustainability because frequent, severe adverse reactions may lead to non-adherence to MDA. If high proportions of the infected or at-risk persons do not take treatment, eliminating the disease may not be achieved [20,25,37,38].

As studies on the safety of LF medicines are limited within the SSA region, and none had been conducted in Kenya, it is important that the NTD program continuously monitors the safety of DA in the population. At the time of this study, Kenya had submitted 11,369 Individual Case Safety Reports to the WHO Global Data Base. However, none of these reports were related to medicines used to treat NTDs through the MDA. Therefore, this study serves as a baseline for the country and will be useful in creating strategies for monitoring AEs during MDA interventions.

## 4. Materials and Methods

### 4.1. Study Design, Area, and Population

**Figure 4:**
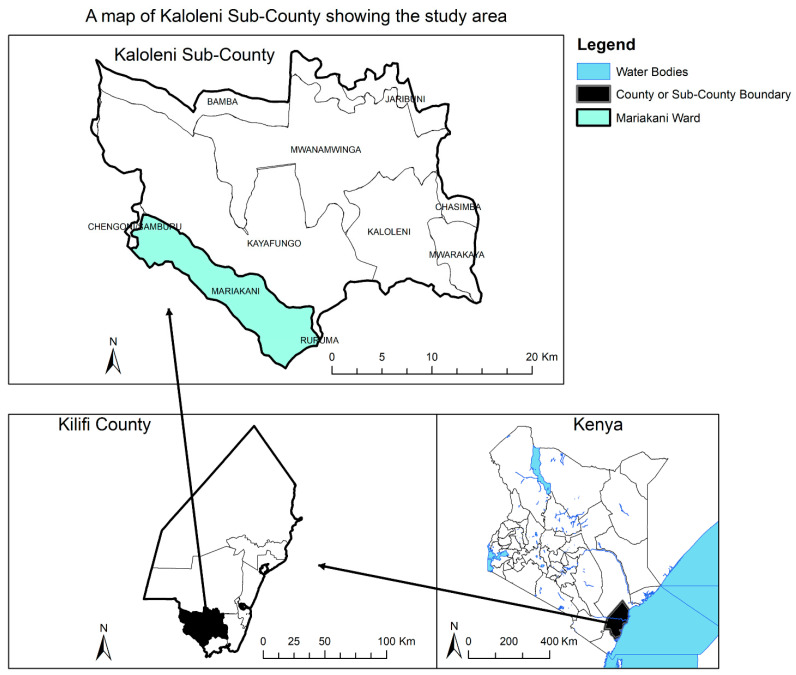
Map of the study area—The map of Kenya located in East Africa (bottom right). The map of Kilifi county (bottom left). Kaloleni Sub county (top), where Mariakani ward, the study site, is located.

This community-based prospective safety surveillance (active cohort event monitoring) was conducted during November 2018–December 2018 in Mariakani Ward, Kaloleni Sub-county of Kilifi County in Kenya (Figure 4). Mariakani, a semi-urban area, was chosen as a study site to enable easy recruitment of large cohort size and allow for easy follow up due to the closeness of the households. Kilifi County is one of the five counties that border the Kenyan coast to the Indian Ocean, covering 12,246 Km^2^, and has seven sub-counties divided into 35 electoral wards. The county population was estimated to be 1,498,647 at the time of this study, as projected from the Kenya Population and Housing Census of 2009, composed of 723,204 males and 775,443 females [39].

### 4.2. Inclusion Criteria

As per the WHO and Kenyan NTD program guideline, any person aged 2 years and above living in LF endemic region is eligible to receive MDA to prevent and control LF [9]. Pregnant women, children aged <2 years, and the severely ill are ineligible populations to receive MDA and were hence excluded from the study. All consenting residents of selected villages who were eligible to receive DEC and ALB preventive chemotherapy according to the national NTD program of Kenya were eligible for enrolment and participation in this study [9,40,41].

### 4.3. Study Sample Size

The sample size was determined based on the WHO’s estimation that a cohort of 3000 individuals gives a 95% probability of identifying a single rare adverse event with an incidence of 1:3000. At-least three events are needed to alert to a signal. Thus, a cohort of 10,000 participants gives a 95% chance of identifying unique or rare events with the incidence rate of 1:3000, based on WHO recommendation for identifying rare drug adverse events [42].

### 4.4. Mass Drug Administration (Preventive Chemotherapy)

This study was open-label and the assignment of eligible subjects to treatments occurred sequentially after initial screening at each site. For proper identification, participants were given identification numbers according to their order of entry into the study. Study participants were interviewed to collect socio-demographic data, anthropometric measures including weight and height, baseline clinical characteristics such as comorbidities, concomitant medications, and LF’s clinical manifestations using a standard case record form (CRF). Furthermore, the presence or absence of any symptoms or events and the type and severity grade were recorded by the data collectors using CRFs before drug administration. Participants received single-dose ALB (400 mg) plus DEC (6 mg/kg) using age as a criterion for DEC dosing following the WHO and the Kenyan national preventive chemotherapy guideline for LF [9,40,41].

### 4.5. Data Collection Procedures and Adverse Events Monitoring

At the time of the study, the Kenyan NTD program projected the ward to have a minimum of 55,063 persons expected to participate in the MDA. The study involved 330 data collectors recruited from the community and trained on how to use the CRFs and document the reported adverse events. For easy access to the community and the households, data collectors were paired with 110 community drug distributors (CDDs) working for the national NTD program to administer the MDA drugs. On the day of the MDA, the survey was conducted through door-to-door visits by a total of 110 teams; each team consisting of one CDD, 1 data collector, and a village elder (Balozi) to assist with community mobilization in each MDA implementation unit (the entire population in an area where LF transmission occurs). Community health extension workers (CHEWs) acted as supervisors for the data collection team. All treated individuals were interviewed for occurrences of any post-MDA AEs actively at 24 h, 48 h, and on day 7 using a standardized questionnaire through a door-to-door follow-up visit. Study participants were advised to contact data collectors and report any suspected AEs they may have experienced between days 3 to 6.

### 4.6. Data Management

Care was taken to ensure the privacy and confidentiality of the information obtained from the study participants. The filled questionnaires and recorded information were stored under lock and key in a designated room; the entered electronic data were stored in password-protected spreadsheets. The data were doubled entered, cleaned, verified, and 10% of the collected data used for data quality assurance to ensure accuracy in the data entry. All reported post-MDA events were cross-checked with events reported before MDA, and only new events following MDA were considered as AEs. Severity grading of AEs was conducted according to the Common Terminology Criteria for Adverse Events (CTCAE) Version 5.0 [43].

- Grade 1 Mild; asymptomatic or mild symptoms; clinical or diagnostic observations only; intervention not indicated.
- Grade 2 Moderate; minimal, local, or non-invasive intervention indicated; limiting age-appropriate Instrumental Activities of Daily Living (ADL).
- Grade 3 Severe or medically significant but not immediately life-threatening; hospitalization or prolongation of hospitalization indicated; disabling; limiting self-care ADL.
- Grade 4 Life-threatening consequences; urgent intervention indicated.
- Grade 5 Death related to AE.

### 4.7. Statistical Analysis

The collected data were analyzed for incidence, types, and predictors of adverse events. Descriptive statistics such as frequencies and proportions were used to summarize categorical variables, while appropriate measures of central tendency and dispersion were used for continuous variables. Bivariate and multivariate log-binomial regression analyses were conducted to measure the association between the predictor variables and adverse events (outcome). The effect of associations was analyzed and presented using risk ratio and adjusted risk ratio with 95% confidence intervals (CIs). Variables with a *p*-value < 0.05 from the bi-variable log-binomial regression analysis were included in the multi-variable log-binomial models. Data were analyzed using STATA version 15.0 software, and *p* < 0.05 was considered as significant.

## 5. Conclusions

Adverse events following mass DEC and ALB co-administration are frequent, occurring one in six of the study population. Post-MDA AEs following DA are systemic, mild-to-moderate, and transient that resolve with 72 h of drug intake. Concomitant medications, chronic illnesses, taking ≥3 tablets of DEC, and being elderly are risk factors for developing AEs. Generally, the co-administration of DEC and ALB (DA) is generally safe and well-tolerated. However, continued AEs monitoring and reporting during the routine MDAs is encouraged to ensure that medicines administered to the public are safe. The study provides important information for national and regional policymakers to minimize medicine-related harm and boost public confidences to achieve the goal of eliminating LF as a public health problem in Kenya.

## Figures and Tables

**Table 1:** Socio-demographic and baseline characteristics of study participants.

Variable		Frequency *n* (%)
Sex	Female	5343 (53.4)
	Male	4667 (46.6)
Age in Years	2 to 15	4456 (44.5)
	16–20	1315 (13.1)
	21–64	3949 (39.5)
	65–99	290 (2.9)
Body Mass Index	Underweight	1898 (19.0)
	Normal	3145 (31.5)
	Overweight	2239 (22.5)
	Obese	2693 (27.0)
Concomitant Medication	Yes	436 (4.4)
	No	9574 (95.7)
Received DA during MDA in the Previous Year (2017)	Yes	5875 (62.3)
	No	3572 (37.8)
Slept Under a Bed net the Previous Night	Yes	9296 (93.5)
	No	642 (6.5)
House Has Mosquito Mesh Screen on Windows	Yes	4800 (48.4)
	No	5127 (51.7)
Indoor Spraying to Prevent Mosquitoes	Yes	3184 (32.2)
	No	6703 (67.8)
Number of DEC Tablets Given	1	1721 (17.2)
	2	2383 (23.8)
	3	5880 (58.7)
	4	26 (0.3)
Chronic Illness	Yes	82 (0.8)
	No	9928 (99.2)

DA = Diethylcarbamazine + Albendazole, DEC = diethylcarbamazine, MDA = mass drug administration.

**Table 2:** Severity grading of adverse events following mass diethylcarbamazine and albendazole administration for control of lymphatic filariasis.

Adverse Events	Total Number of Events	Severity Grading		
		Grade 1 (Mild)	Grade 2 (Moderate)	Grade 3 (Severe)
Dizziness	550	489 (88.9%	60 (10.9%)	1 (0.2%)
Headache	530	426 (80.4%)	97 (18.3%)	7 (1.3%)
Loss of App	312	288 (92.3%)	24 (7.7%)	
Drowsiness	245	222 (90.6%)	23 (9.4%)	
Fever	239	218 (91.2%)	21 (8.8%)	
Cough	208	187 (89.9%)	21 (10.1%)	
Nausea	185	163 (88.1%)	21 (11.4%)	1 (0.5%)
Stomach Pain	183	156 (85.2 %)	27 (14.8%)	
Vomiting	175	144 (82.3 %)	31 (17.7%)	
Confusion	98	93 (94.9 %)	5 (5.1%)	
Diarrhoea	103	86 (83.5 %)	17 (16.5%)	
Difficulty in Breathing	37	30 (81.1 %)	7 (18.9%)	
Total	2865	2502 (87.3%)	354 (12.4%)	9 (0.3%)

**Table 3:** Incidence and correlations of adverse events following mass diethylcarbamazine and albendazole administration for the elimination of lymphatic filariasis.

Variables		Adverse Event		X^2^	*p*-Value
		No (*n* = 8389)	Yes (*n* = 1621)		
Sex	Male	3927 (84.14)	740 (15.86)	0.74	0.39
	Female	4462 (83.51)	881 (16.49)		
Age in Years	2–15 years	3795 (85.17)	661 (14.83)	24.50	<0.001
	16 to 20	1056 (80.30)	259 (19.70)		
	21–64	3311 (83.84)	638 (16.16)		
	65–99	227 (78.28)	63 (21.72)		
Taking Concomitant Medication	Yes	323 (74.08)	113 (25.92)	31.76	<0.001
	No	8066 (84.25)	1508 (15.75)		
Received DA During MDA in the Previous Year (2017)	Yes	4911 (83.59)	964 (16.41)	0.57	0.45
	No	3007 (84.18)	565 (15.82)		
Slept Under a Bed Net the Previous Night	Yes	7818 (84.1)	1478 (15.9)	11.62	0.001
	No	507 (78.97)	135 (21.03)		
Availability of Screen on the Windows	Yes	4093 (85.27)	707 (14.73)	15.17	<0.001
	No	4224 (82.39)	903 (17.61)		
Indoor Spraying to Prevent Mosquitoes	Yes	2659 (83.51)	525 (16.49)	0.14	0.70
	No	5618 (83.81)	1085 (16.19)		
Number of DEC Tablets Given	1	1488 (86.46)	233 (13.54)	18.52	<0.001
	2	2026 (85.02)	357 (14.98)		
	3	4854 (82.55)	1026 (17.45)		
	4	21 (80.77)	5 (19.23)		
Chronic illness	Yes	62 (75.61)	20 (24.39)	4.09	0.04
	No	8327(83.87)	1601(16.13)		
Type of Meal	Carbohydrate	4436(85.79)	735 (14.21)	20.60	<0.001
	Fatty	751(82.44)	160(17.56)		
	High protein	604 (80.11)	150(19.89)		

**Table 4:** Predictors of adverse events following mass diethylcarbamazine and albendazole administration for the elimination of lymphatic filariasis.

Variables		Crude Risk Ratios	*p*-Value	95% CI	Adjusted Risk Ratios	*p*-Value	95% CI
Sex	Female	1			1		
	Male	0.96	0.39	0.88–1.05			
Age in Years	2 to 15	0.92	0.09	0.83–1.01	1.19	0.07	0.99–1.42
	16 to 20	1.22	0.003	1.07–1.39	1.22	0.02	1.03–1.45
	21 to 64	1			1		
	65 to 99	1.34	0.01	1.07–1.69	1.26	0.14	0.93–1.70
Taking Concomitant Medication	Yes	1.65	<0.001	1.39–1.94	1		
	No	1			1.82	<0.001	1.48–2.25
Received DA During MDA in the Previous Year (2017)	No	0.96	0.45	0.88–1.06			
	Yes	1					
Number of DEC Tablets Given	1	1					
	2	1.11	0.20	0.95–1.29	1.08	0.39	0.90–1.30
	≥3	1.29	<0.001	1.13–1.47	1.24	0.05	1.00–1.53
Chronic Illness	Yes	1.51	0.04	1.03–2.22	1.18	0.46	0.76–1.84
	No	1			1		
Type of Meal before MDA	Carbohydrate	1			1		
	Fatty	1.24	0.008	1.06–1.44	1.22	0.01	1.04–1.42
	High protein	1.40	<0.001	1.19–1.64	1.40	<0.001	1.19–1.63

DEC = diethylcarbamazine, LF = lymphatic Filariasis, MDA = mass drug administration, CI=confidence interval.

## Data Availability

All data presented in this study are contained within the manuscript.
